# Cerebral responses to vocal attractiveness and auditory hallucinations in schizophrenia: a functional MRI study

**DOI:** 10.3389/fnhum.2013.00221

**Published:** 2013-05-24

**Authors:** Michihiko Koeda, Hidehiko Takahashi, Masato Matsuura, Kunihiko Asai, Yoshiro Okubo

**Affiliations:** ^1^Voice Neurocognition Laboratory, The Centre for Cognitive Neuroimaging, The Institute of Neuroscience and Psychology, University of GlasgowGlasgow, UK; ^2^Department of Neuropsychiatry, Nippon Medical SchoolTokyo, Japan; ^3^Department of Psychiatry, Kyoto UniversityKyoto, Japan; ^4^Department of Biofunctional Informatics, Tokyo Medical and Dental UniversityTokyo, Japan; ^5^Asai HospitalChiba, Japan

**Keywords:** attractiveness, auditory hallucinations, schizophrenia, greeting, cerebral laterality, social communications, functional MRI

## Abstract

Impaired self-monitoring and abnormalities of cognitive bias have been implicated as cognitive mechanisms of hallucination; regions fundamental to these processes including inferior frontal gyrus (IFG) and superior temporal gyrus (STG) are abnormally activated in individuals that hallucinate. A recent study showed activation in IFG-STG to be modulated by auditory attractiveness, but no study has investigated whether these IFG-STG activations are impaired in schizophrenia. We aimed to clarify the cerebral function underlying the perception of auditory attractiveness in schizophrenia patients. Cerebral activation was examined in 18 schizophrenia patients and 18 controls when performing Favorability Judgment Task (FJT) and Gender Differentiation Task (GDT) for pairs of greetings using event-related functional MRI. A full-factorial analysis revealed that the main effect of task was associated with activation of left IFG and STG. The main effect of Group revealed less activation of left STG in schizophrenia compared with controls, whereas significantly greater activation in schizophrenia than in controls was revealed at the left middle frontal gyrus (MFG), right temporo-parietal junction (TPJ), right occipital lobe, and right amygdala (*p* < 0.05, FDR-corrected). A significant positive correlation was observed at the right TPJ and right MFG between cerebral activation under FJT minus GDT contrast and the score of hallucinatory behavior on the Positive and Negative Symptom Scale. Findings of hypo-activation in the left STG could designate brain dysfunction in accessing vocal attractiveness in schizophrenia, whereas hyper-activation in the right TPJ and MFG may reflect the process of mentalizing other person's behavior by auditory hallucination by abnormality of cognitive bias.

## Introduction

Auditory hallucinations and thought disorder are the main symptoms of schizophrenia, and these symptoms profoundly affect the neural basis of social communications as well as behavior (Brune et al., 2008; Bucci et al., 2008; Wible et al., 2009; Kumari et al., 2010; Granholm et al., 2012; Waters et al., 2012). In order to understand these psychiatric symptoms in schizophrenia, it is important to verify the pathophysiology of cerebral function in auditory communications.

For healthy people, greeting conversations are very essential tools for communicating socially with family, friends, and community. Since favorable greetings strengthen cordial relationships with colleagues, maintaining the skill of socializing with greeting conversations is especially significant (Gronna et al., 1999; Barry et al., 2003). One of the main cognitive models in schizophrenia proposes that hallucinations arise from impaired self-monitoring and abnormality of cognitive bias (Allen et al., 2004). Some studies indicate that schizophrenia patients tend to misapprehend inner speech as external speech by the disturbance of self-monitoring (Morrison and Haddock, 1997; Stein and Richardson, 1999; Ford et al., 2001; Allen et al., 2004). A recent study has suggested that auditory hallucination in schizophrenia may be caused by both impaired brain function in auditory processing and disturbance of attention bias toward internally generated information (Kompus et al., 2011). If patients with schizophrenia mistake unfavorable greetings through their distorted thinking while listening to favorable greetings, social isolation and emotional withdrawal could be produced. In addition, if schizophrenia patients have auditory hallucination, misjudgment of favorable/unfavorable greeting may be induced by abnormality of cognitive bias. However, it is unclear whether schizophrenia patients with auditory hallucinations have impaired abilities to differentiate between favorable and unfavorable greetings.

Functional magnetic resonance imaging (fMRI) studies in schizophrenia have investigated the neural basis of impairment of paralinguistic processing such as emotional prosody and affective vocalizations (Mitchell et al., 2004; Leitman et al., 2007, 2011; Bach et al., 2009; Dickey et al., 2010) as well as language processing (Woodruff et al., 1997; Kircher et al., 2001; Mitchell et al., 2001; Sommer et al., 2001; Schettino et al., 2010). A previous fMRI study concerning the recognition of emotional speech prosody demonstrated that temporal activation in schizophrenia patients was predominant in the left hemisphere, whereas that in normal control subjects showed right hemispheric dominance (Mitchell et al., 2004). Another fMRI study also found right-lateralized activation in healthy controls in the temporal-parietal region while listening to emotional prosody including meaningless syllables (Bach et al., 2009). In schizophrenia patients, however, this right-lateralized pattern was even more pronounced. These findings indicate that cerebral laterality for emotional prosody in schizophrenia patients could be shifted in comparison to the typical right-lateralized activation in normal control subjects.

We consider that it is important to investigate the relationship between psychiatric symptom and cerebral function in behavior social as well as emotional prosody. Especially, evaluating facial attractiveness is a favorable behavior associated with social communication (Kampe et al., 2001; Winston et al., 2007). Recent studies have demonstrated that facial attractiveness can activate doperminergic regions including amygdala and orbitofrontal cortex that are strongly related to reward prediction (Winston et al., 2007; Cloutier et al., 2008; Chatterjee et al., 2009; Tsukiura and Cabeza, 2011). Clarifying brain mechanisms in these reward systems is very important for understanding the pathophysiology of schizophrenia. A recent study has shown that in schizophrenia, the ratings of attractiveness of unfamiliar faces were significantly reduced compared to healthy subjects (Haut and MacDonald, 2010). Further, this study has demonstrated that when the patient had severe persecutory delusions, attractiveness ratings decreased (Haut and MacDonald, 2010). As well as facial perception, auditory attractiveness in schizophrenia will be a challenging research topic. A recent fMRI study in healthy subjects on auditory attractiveness has demonstrated bilateral superior temporal gyrus (STG) and inferior frontal gyrus (IFG) activates when participants judged whether voices sounded attractive or not. This study suggests that the roles of STG and IFG are essential for perceiving auditory attractiveness (Bestelmeyer et al., 2012). The regions of STG and IFG are heavily implicated in the functional anatomy of auditory hallucination. A recent meta-analysis demonstrated that schizophrenia patients with auditory hallucination had significantly increased activity in fronto-temporal areas involved in speech generation and speech perception (Jardri et al., 2011). A recent fMRI study demonstrated that cerebral activation in fronto-temporal regions is greater than in healthy individuals during AVH but lower during environmental-stimulus processing (Kompus et al., 2011). However, to our knowledge, no study has ever investigated the cerebral response to auditory attractiveness in schizophrenia.

The aim of our research is to clarify cerebral response to auditory attractiveness when patients with schizophrenia are listening to greetings. Greeting conversations are crucial to maintaining social interactions. An fMRI study of social perception indicated that the left prefrontal and left IFG were activated when the subjects judged whether two people were friends or enemies (Farrow et al., 2011). Since the recognition of friendliness and favorability is essential for greeting conversations, the patients with schizophrenia could change cerebral function due to psychiatric symptoms such as auditory hallucinations. To investigate this pathophysiology, using completely the same greetings, we compared cerebral activation when the subjects judged favorability (recognition of auditory attractiveness) and cerebral activation when the subjects judged gender (recognition of non-auditory attractiveness). Prior to the current experiment, we hypothesized that cerebral functions underlying the perception of auditory attractiveness could be impaired in STG and IFG by occurring auditory hallucination.

## Materials and methods

### Subjects of fMRI study

Eighteen right-handed controls (9 males and 9 females, mean age 35.5 years, *SD* = 8.6) and 18 schizophrenia patients (10 males and 8 females, mean age 35.7 years, *SD* = 8.4) participated in the present study. As for the subtypes of 18 schizophrenia patients, all patients were diagnosed with paranoid schizophrenia. All 18 patients were receiving neuroleptics (mean risperidone equivalent daily dosage = 4.7 mg, *SD* = 2.2; 9 patients, risperidone; 4 patients, olanzapine; 2 patients, haloperidol; 1 patient, quetiapine; 1 patient, sulpiride; 1 patient, perphenazine). Risperidone equivalents were calculated based on published equivalencies for atypical antipsychotics by Inagaki and Inada (2006). All 36 volunteers were native speakers of Japanese. None of the control subjects was taking alcohol or medication at the time, nor did they have a history of psychiatric disorder, significant physical illness, head injury, neurological disorder, or alcohol or drug dependence. After complete explanation of the study, written informed consent was obtained from all subjects, and the study was approved by the relevant ethics committee. Schizophrenia patients were diagnosed by MK and the attending psychiatrists on the basis of a review of their charts and a conventionally semi-structured interview (First et al., 1995). After the structural interview was performed using PANSS, the patient was synthetically diagnosed according to the diagnostic guidelines of the ICD-10: Classification of Mental and Behavioral Disorders. Exclusion criteria were current or past substance abuse and a history of alcohol-related problems, mood disorder, or organic brain disease. All patients were recruited from the outpatient unit of Asai Hospital. Mean illness duration was 12.3 (*SD* = 8.0) years. Clinical symptoms were assessed by Positive and Negative Syndrome Scale (PANSS) (Kay et al., 1987). Sum scores for positive and negative symptoms were calculated, with the positive symptom subscale including the following seven items: Delusion, Conceptual disorientation, Hallucinatory behavior, Excitement, Grandiosity, Suspiciousness, and Hostility. The negative symptom subscale also included seven items: Blunted affect, Emotional withdrawal, Poor rapport, Passive/apathetic social withdrawal, Difficulty in abstract thinking, Lack of spontaneity and flow of conversation, and Stereotyped thinking. The mean score of PANSS was 32.4 (*SD* = 10.4). The mean positive symptom score was 15.1 (*SD* = 6.4), mean negative symptom score was 20.7 (*SD* = 6.2), and mean score of general psychopathology was 32.3 (*SD* = 7.9). The candidates were carefully screened and standardized interviews were conducted by a research psychiatrist (MK) and the attending psychiatrists. They did not meet the criteria for any psychiatric disorders. There was no significant difference in the mean period of education between the controls and patients (mean ± *SD*; patients 13.3 ± 1.3 years, control subjects 13.0 ± 1.0 years; *p* > 0.05, *t*-test). Schizophrenia patients were 14 right-handed and 4 left-handed participants according to the Edinburgh Handedness Inventory (EHI) (Oldfield, 1971). Mean (± *SD*) EHI in right-handed 14 patients was 90.4 ± 13.0. The EHI score of the 4 left-handed patients was −85, −73, −46, −46, respectively. All control subjects were right-handed, and mean (± *SD*) EHI was 96.1 ± 4.7.

### Recorded voice

As a sample for clarifying emotional response in voice recognition, Japanese greetings were recorded from 6 native speakers (3 males, 3 females). Ten greetings were recorded: Ohayo (Good Morning), Yah (Hi), Konnichiwa (Good Afternoon), Konbanwa (Good evening), Arigato (Thank you), Domo (Thank you), Irasshai (Welcome), Genki (How are you?), Dozo (Please), and Hisashiburi (Long time no see). These 10 greetings were recorded expressing favorable emotion (positive greeting), unfavorable emotion (negative greeting), or without emotion (neutral greeting), resulting in 180 stimuli in total. The voice was recorded using an IC recorder (Voice-Trek DS-71, Olympus) in a perfectly quiet room. In both the preliminary experiment and the fMRI experiment, all speakers were unknown to all participants.

### Preliminary examination

Prior to the fMRI study, we asked 32 different control volunteers (16 males and 16 females) to judge the favorability of all 180 greetings (60 favorable, 60 non-favorable, and 60 neutral greetings) using a questionnaire with a 10-point scale. We defined “favorable” if the scale approached 10, whereas “unfavorable” if the scale approached 0. Based on the responses of the 32 subjects, greetings were considered positive if their average score was higher than 6.5. If the average score was less than 3.5, the greetings were considered negative. Neutral greetings were defined as being located within the average score range of 4.5–5.5. Based on these results, each speaker's greeting was evenly selected for the favorable, neutral, and unfavorable greetings.

### Instruments used for presentation of stimuli

Stimuli were presented by the use of Media Studio Pro (version 6.0 Ulead Systems, Inc., Ulead Systems, Taiwan) running under Windows XP. Subjects listened to the sound stimuli through headphones attached to an air conductance sound delivery system (Commancer X6, MRI Audio System, Resonance Technology Inc., Los Angeles, CA). The average sound pressure of stimulus amplitude was kept at 80 dB.

### Experiment design

**Figure 1:**
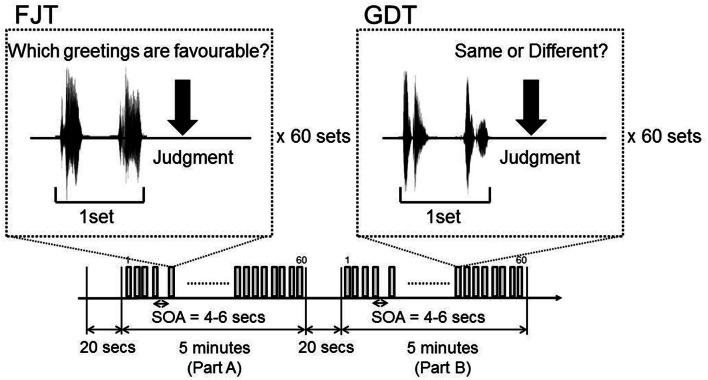
**Experimental design of fMRI Part A is Favorability Judgment Task (FJT).** Part B is Gender Discrimination Task (GDT).

The subjects listened for a total of 10 min and 40 s: 20 s of silence, 5 min of attentive listening (Part A), 20 s of silence, and 5 min of attentive listening (Part B). Part A and Part B each consisted of 60 paired greetings (30: neutral-positive, 30 neutral-negative), with each greeting taking 0.5 s, and pause of 1 s; all together, each of the 10 greetings was spoken 12 times (6 times as a neutral greeting, 3 times as a positive greeting, and 3 times as a negative greeting). In Part A, there were equal numbers of greetings by male pairs and female pairs. The subjects judged which greeting of a pair was more favorable. Using 30 neutral-positive and 30 neutral-negative pairs, we examined the degree of difference in favorability. We named Part A: Favorability Judgment Task (FJT). In Part B, 30 pairs were the same gender and 30 pairs were different gender. The subjects judged whether the speakers in each pair were the same gender or not. We named Part B: Gender Discrimination Task (GDT). The pairings of neutral-positive and neutral-negative appeared in random order (Figure 1).

### Functional MRI acquisition

The images were acquired with a 1.5 Tesla Signa system (General Electric, Milwaukee, Wisconsin). Functional images of 264 volumes were acquired with T2^*^-weighted gradient echo planar imaging sequences sensitive to blood oxygenation level dependent (BOLD) contrast. Each volume consisted of 20 transaxial contiguous slices with a slice thickness of 6 mm to cover almost the whole brain (flip angle, 90°; time to echo [TE], 50 ms; repetition time [TR], 2.5 s; matrix, 64 × 64; field of view, 24 × 24).

### Image processing

Data analysis was performed with statistical parametric mapping software SPM8 (Wellcome Department of Cognitive Neurology, London, United Kingdom) running with MATLAB (Mathworks, Natick, Massachusetts). All volumes of functional EPI images were realigned to the first volume of each session to correct for subject motion, and the mean functional EPI image was spatially coregistered with the anatomical T1 images. The anatomical T1 image was segmented into the image of gray matter and white matter. Based on the segmented T1 image of each subject, the anatomical template of diffeomorphic anatomical registration through an exponentiated Lie algebra (DARTEL) was created (Ashburner, 2007). All realigned EPI images were spatially normalized to the standard space defined by the Montreal Neurological Institute (MNI) template with DARTEL template and flow field of each subject. Functional images were spatially smoothed with a 3-D isotropic Gaussian kernel (full width at half maximum of 8 mm). A temporal smoothing function was applied to the fMRI time series to enhance the temporal signal-to-noise ratio. The significance of hemodynamic changes in each condition was examined using the general linear model with boxcar functions convoluted with a hemodynamic response function. The *t*-values were then transformed to unit normal distribution, resulting in z-scores. The models of 4 contrasts were created by event-related design during the fMRI experiments. In FJT task, 2 contrasts [30 pairs of neutral-favorable greetings (FAV) and 30 pairs of neutral-unfavorable greetings (NFV)] were made. In GDT task, 2 contrasts [30 pairs of same gender greetings (SAM) and 30 pairs of different gender greetings (DIF)] were made (Figure 1).

### Statistical analysis

Group analysis (2nd-level analysis in spm8) was performed on the data for 18 control subjects and 18 schizophrenia patients using a random effect model on a voxel-by-voxel basis. FMRI data was analysed based on the 2 × 2 × 2 full factorial model with the factors of Group (control subjects/schizophrenia patients), Task (FJT/GDT) and Within-task (FJT: FAV/NFV, GDT: SAM/DIF) (FDR-corrected voxel-level threshold of *P* < 0.05). By using rfxplot (Glascher, 2009), cerebral activation at the regions of interests (ROIs) was investigated. In main effect of Group and main effect of Task, ROIs were focused on the coordinates of the peak voxel of activation under FDR-corrected voxel-level threshold of *P* < 0.05. For main effect of Group, cerebral laterality of ROIs was evaluated. In order to investigate cerebral laterality, ROIs were also set on the hemispheric symmetrical region of the MNI coordinates. By using these symmetrical ROIs, the laterality index (LI) was calculated [LI = (L − R)/(L + R) × 100; L = beta estimates of left hemispheric activation, R = beta estimates of right hemispheric activation]. The formula of the LI was calculated based on previous studies (Koeda et al., 2006, 2007; White et al., 2009). In calculation of LI, the beta value of each subject used was either plus or zero, and minus beta values were excluded. In this ROI analysis, correlation between EHI score and beta value was evaluated to investigate the influence of handedness. Correlations between the subscores of PANSS (total scores of positive symptoms, negative symptoms, and general psychopathology) and cerebral activation under FJT vs. GDT contrast were calculated based on simple regression in schizophrenia patients. In linear regression analyses, the three subscores of PANSS were used, each with one predictor, respectively. In the analysis of full factorial design, the statistical threshold used was *p* < 0.05, voxel level, FDR-corrected. In the linear regression analysis, the statistical threshold used was *p* < 0.0001, voxel level, uncorrected (FDR < 0.25, voxel level corrected). Further, the correlation was analysed between the beta value of FJT at the specific ROIs of main effect of Group (Figure 10).

## Results

### Preliminary experiments

**Figure 2:**
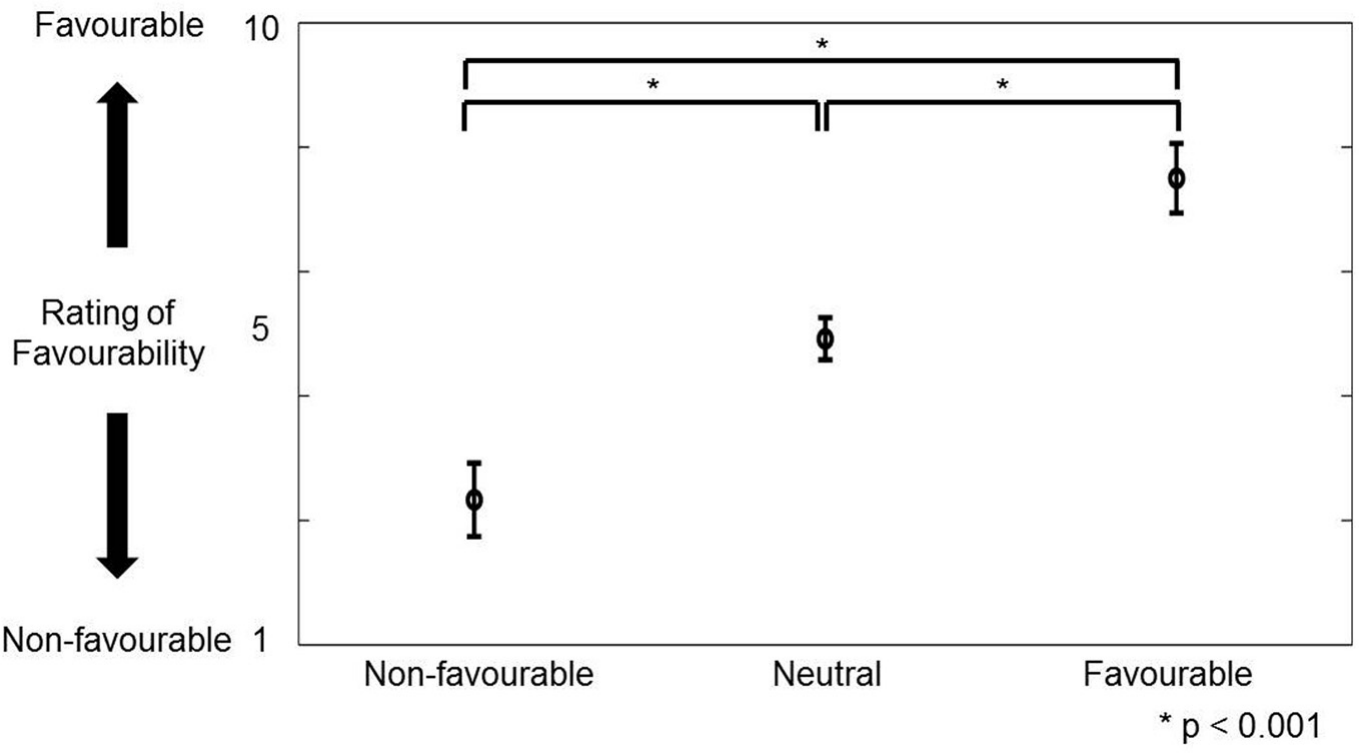
**The figure shows the distribution (mean ± *SD*) of rating favorability for portraying favorable, neutral, and unfavorable greetings in preliminary experiment.** The vertical axis represents the rating score of favorability (1–10). ^*^Indicates that multiple comparison was significant.

Favorability was rated by 32 different control volunteers using a scale of 1–10. Figure 2 shows the distribution of the rating of favorability. Based on the definition of favorability (Materials and Methods: Preliminary Examination), 30 favorable vocalizations (rating average more than 6.5; 12 males and 9 females), 60 neutral vocalizations (rating average between 4.5 and 5.5; 17 males and 18 females), 30 unfavorable vocalizations (rating average less than 3.5; 7 males and 13 females) were selected. The mean ratings (± *SD*) of favorability were 2.3 ± 0.6 (unfavorable), 4.9 ± 0.3 (neutral), and 7.5 ± 0.6, respectively. Analysis of variance (One-Way ANOVA) was significantly different [*F*_(2, 117)_ = 1006.9, *p* < 0.001]. Multiple comparisons were also significant (unfavorable vs. neutral: 2.6 ± 0.1, *p* < 0.001; neutral vs. favorable: 2.6 ± 0.1, *p* < 0.001; unfavorable vs. favorable: 5.2 ± 0.1, *p* < 0.001).

#### Behavioral data (accuracy)

**Figure 3:**
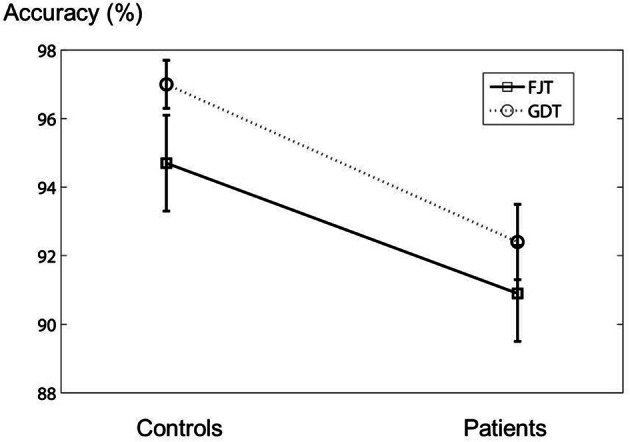
**The figure shows the error bar (mean ± *SD*) of the accuracy in the fMRI experiment.** The vertical axis represents the accuracy of the experiment.

In the fMRI experiment, the mean percentages (±*SD*) of the accuracy of the control subjects for FJT and GDT were 94.7 ± 6.1% and 97.0 ± 3.1%, and those of schizophrenia patients were 90.9 ± 6.0% and 95.1 ± 4.8%, respectively (Figure 3). There was no significant difference between the two groups [FJT: *t*_(34)_ = 1.89, *p* > 0.05; GDT: *t*_(34)_ = 1.45, *p* > 0.05]. Mixed analysis of variance (mixed ANOVA) in the performance did not show a significant main effect of Group (control subjects/schizophrenia patients): *F*_(1, 34)_ = 2.33, *p* > 0.05, whereas a significant Task effect (FJT vs. GDT) was observed: *F*_(1, 34)_ = 11.5, *p* < 0.001. No interaction effect between Group and Task was observed: *F*_(1, 34)_ =0.19, *p* > 0.05. Table 1 shows the mean accuracy for judgment of favorable/non-favorable, and judgment of same gender and different gender (FAV controls: 93.5 ± 7.6%; FAV patients: 94.1 ± 6.2%; NFV controls: 94.7 ± 6.1%; NFV patients: 90.0 ± 6.0%; SAM controls: 98.3 ± 2.4%; SAM patients: 95.7 ± 5.6%; DIF controls: 97.2 ± 3.8%; DIF patients: 95.0 ± 4.2%). No significant difference was observed between controls and patients [FAV: *t*_(34)_ = −0.24, *p* > 0.05; NFV: *t*_(34)_ = 1.89, *p* > 0.05; SAM: *t*_(34)_ = 1.82, *p* > 0.05; DIF: *t*_(34)_ = 1.67, *p* > 0.05]. Three-Way ANOVA was calculated for the effect of Group, Task, and Within-task. Task effect was significantly observed [*F*_(1, 34)_ = 11.9, *p* = 0.002], whereas Group effect and Within-task effect were not observed [Group: *F*_(1, 34)_ = 2.33, *p* > 0.05; Within-task: *F*_(1, 34)_ = 2.55, *p* > 0.05]. Interaction effect was not observed in the effect of Group × Task [*F*_(1, 34)_ = 0.17, *p* > 0.05] and the effect of Group × Within-task [*F*_(1, 34)_ = 2.80, *p* > 0.05].

**Table 1:** **Shows the mean ± *SD* of accuracy and response time in fMRI experiments**.

		**FAV**	**NFV**	**SAM**	**DIF**
Accuracy (%)	Controls	93.5 ± 7.6	94.7 ± 6.1	98.3 ± 2.4	97.2 ± 3.8
	Patients	94.1 ± 6.2	90.9 ± 6.0	95.7 ± 5.6	95.0 ± 4.2
Response time (sec)	Controls	2.41 ± 0.78	2.01 ± 0.13	2.15 ± 0.78	1.99 ± 0.53
	Patients	2.17 ± 0.17	1.89 ± 0.15	1.93 ± 0.14	2.07 ± 0.13

*FAV, pairs of neutral-favorable greetings; NFV, pairs of neutral-unfavorable greetings; SAM, pairs of same gender greetings; DIF, pairs of different gender greetings*.

#### Response time

The mean (± *SD*) response times relative to offset of stimulus (seconds) of control subjects and schizophrenia patients for FJT and GDT were the following: FJT-control: 2.41 ± 0.78 s, FJT-patients: 2.01 ± 0.33 s, GDT-control: 2.15 ± 0.78 s, and GDT-patients 1.99 ± 0.05 s. The response times in FJT were significantly different between control subjects and schizophrenia patients: *t*_(34)_ = 2.16, *p* = 0.04 < 0.05, whereas there was no significant difference in the response times to GDT: *t*_(34)_ = 0.84 *p* > 0.05. Analysis of variance on the response time was performed with the factors of Group (control subjects/schizophrenia patients) and Task (FJT/GDT). There was no significant difference between Groups: *F*_(1, 34)_ = 2.29, *p* > 0.05, whereas there was a significant Task effect: *F*_(1, 34)_ = 33.7, *p* < 0.001. An interaction effect between Group and Task was also observed: *F*_(1, 34)_ = 24.4, *p* < 0.001. Table 1 (lower part) shows the mean response time for judgment of favorable/non-favorable, and judgment of same gender and different gender [FAV controls: 2.41 ± 0.78 s; FAV patients: 2.17 ± 0.17 s; NFV controls: 2.01 ± 0.13 s; NFV patients: 1.89 ± 0.15 s; SAM controls: 2.15 ± 0.78 s; SAM patients: 1.93 ± 0.14 s; DIF controls: 1.99 ± 0.53 s; DIF patients: 2.07 ± 0.13 s]. Significant difference between controls and patients was observed in NFV and DIF [NFV: *t*_(18.4)_ = 3.49, *p* > 0.05; DIF: *t*_(22.8)_ = −2.31, *p* < 0.05: Welch's *t*-test], whereas no significant difference was observed in FAV and SAM [FAV: *t*_(18.6)_ = 1.29, *p* > 0.05; SAM: *t*_(18.1)_ = 1.15, *p* > 0.05: Welch's *t*-test]. Three-Way ANOVA was calculated for the effect of Group, Task, and Within-task. Task effect was significantly observed [*F*_(1, 34)_ = 36.7, *p* < 0.001], whereas Group effect and Within-task effect were not observed [Group: *F*_(1, 34)_ = 1.93, *p* > 0.05; Within-task: *F*_(1, 34)_ = 0.13, *p* > 0.05]. Interaction effect was significantly observed in the effect of Group × Task [*F*_(1, 34)_ = 16.0, *p* < 0.001], whereas interaction effect was not significantly observed in the effect of Group × Within-task [*F*_(1, 34)_ = 1.04, *p* > 0.05].

### Functional MRI data

#### Full factorial design analysis

FMRI data was analysed based on the 2 × 2 × 2 full factorial model with the three factors: Group (control subjects/schizophrenia patients), Task (FJT/GDT), and Within-task (FJT: FAV/NFV, GDT: SAM/DIF) (FDR-corrected voxel-level threshold of *P* < 0.05).

**Figure 4:**
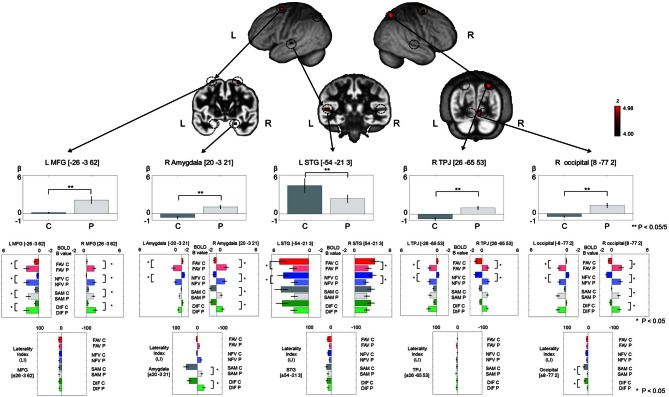
**The figure demonstrates the results of main effect of Group (controls/patients) in analysis of 2 × 2 × 2 full factorial design.** Upper pictures show cerebral activation on main effect of Group (*p* < 0.05, voxel level, FDR-corrected). Gray bars in the upper row represent the distribution of beta values (mean ± SE) in controls (dark gray color) and patients (light gray color). The vertical axis, beta values; C, controls; P, patients; L, left; R, right. ^**^Indicates *p* < 0.05/5 (Bonferroni correction by 5 ROIs). Color bars in the middle row represent the distribution of beta values (mean ± SE) on 5 ROIs and contralateral symmetrical 5 ROIs under FAV (red), NFV (blue), SAM (gray), and DIF (green) conditions—C, controls (dark gray); P, patients (light gray). Color bars at the bottom represent the distribution of laterality index (mean ± SE) on the 5 ROIs. ^*^Indicates *p* < 0.05. MFG, middle frontal gyrus; STG, superior temporal gyrus; TPJ, temporo-parietal junction; occipital, occipital lobe.

Main effect of Group was significantly observed in the bilateral middle frontal gyrus (MFG), left STG, right superior parietal lobe (SPL) temporo-parietal junction (TPJ), right occipital lobe, and right amygdala (*p* < 0.05, FDR-corrected, Figure 4 and Table 2). The upper part (gray bar) of Figure 4 shows the bar graph for contrast estimates and 90% confidence interval in each activated region (gray bar: controls, the light gray bar: patients). From the results of main effect of Group, ROIs were set on the 5 regions: left MFG [−26, −3, 62], right amygdala [20, −3, 21], left STG [−54, −21, 3], right TPJ [26, −65, 53], and right occipital lobe [8, −77, 2]. In these ROIs, Mann–Whitney test was calculated for beta values between controls and patients. The P-threshold was Bonferroni-corrected based on 5 tests being conducted. Cerebral activation in left STG was significantly greater in control subjects than in schizophrenia patients (L STG: *z* = 3.10, *p* = 0.001 < 0.05/5), whereas cerebral activations in the other regions were significantly greater in schizophrenia patients than in control subjects (L MFG: *z* = −3.61, *p* < 0.05/5; R amygdala: *z* = −3.54, *p* < 0.05/5; R TPJ: *z* = −3.61, *p* < 0.05/5; R occipital: *z* = −3.54, *p* < 0.05/5). In these 5 ROIs and contralateral symmetrical 5 ROIs (right MFG [26, −3, 62], left amygdala [−20, −3, 21], right STG [54, −21, 3], left TPJ [−26, −65, 53], left occipital lobe [−8, −77, 2]), cerebral activation under FAV, NFV, SAM, and DIF conditions was evaluated (middle part of Figure 4). Further, the LI was calculated (lower part of Figure 4). For each ROI, Two-Way ANOVA was calculated by main effect of Group and Within-task. Regarding Group effect, in the ROIs at the bilateral MFG, bilateral amygdala, bilateral TPJ, and right occipital lobe, the strength of BOLD signal (beta estimates) in patients under the FAV and NFV conditions was significantly greater than that in controls [L MFG: *F*_(1, 34)_ = 14.1, *p* < 0.001; R MFG: *F*_(1, 34)_ = 21.5, *p* < 0.001; L amygdala: *F*_(1, 34)_ = 14.9, *p* < 0.001; R amygdala: *F*_(1, 34)_ = 18.0, *p* < 0.001; L TPJ: *F*_(1, 34)_ = 12.7, *p* < 0.001; R TPJ: *F*_(1, 34)_ = 4.67, *p* < 0.05; R occipital: *F*_(1, 34)_ = 14.4, *p* < 0.001], whereas that in bilateral STG was significantly greater in controls than in patients [L STG: *F*_(1, 34)_ = 12.7, *p* < 0.001; R STG: *F*_(1, 34)_ = 4.7, *p* < 0.05]. In bilateral MFG, right amygdala, and right occipital, BOLD signals of patients under SAM and DIF conditions were significantly greater than in controls [L MFG: *F*_(1, 34)_ = 14.1, *p* < 0.001; R MFG: *F*_(1, 34)_ = 21.5, *p* < 0.001; R amygdala: *F*_(1, 34)_ = 7.8, *p* < 0.01; R occipital: *F*_(1, 34)_ = 5.9, *p* < 0.05]. Significant difference of LI was observed in the amygdala and occipital lobe under SAM and DIF conditions [amygdala LI: *F*_(1, 34)_ = 7.8, *p* < 0.001; occipital lobe: *F*_(1, 34)_ = 6.5, *p* < 0.05], whereas significant difference in the other regions was not observed (*p* > 0.05).

**Table 2:** **Peak coordinates (*x*, *y*, *z*) and their *z*-values of cerebral activation by full factorial design analysis with Group effect (controls and patients)**.

**Brain regions**	***BA***	**Coordinate**			***F*_(1, 136)_**	***z*-value**	***P* (FDR-corrected)**
		***x***	***y***	***z***			
**MAIN EFFECT OF GROUP (CONTROLS/SCHIZOPHRENIA)**							
Controls > Patients							
L STG	41	−54	−21	3	23.20	4.47	<0.05
Patients > Controls							
L MFG	6	−26	−1	63	23.40	4.49	<0.05
R MFG	6	27	−1	61	19.50	4.10	<0.05
R SPL	7	26	−64	52	29.70	5.04	<0.05
Occipital lobe	18	8	−76	1	21.80	4.34	<0.05
R amygdala		20	−3	−21	18.40	3.99	<0.05

*L, left hemisphere; R, right hemisphere; p < 0.05, voxel level, FDR-corrected*.

**Figure 5:**
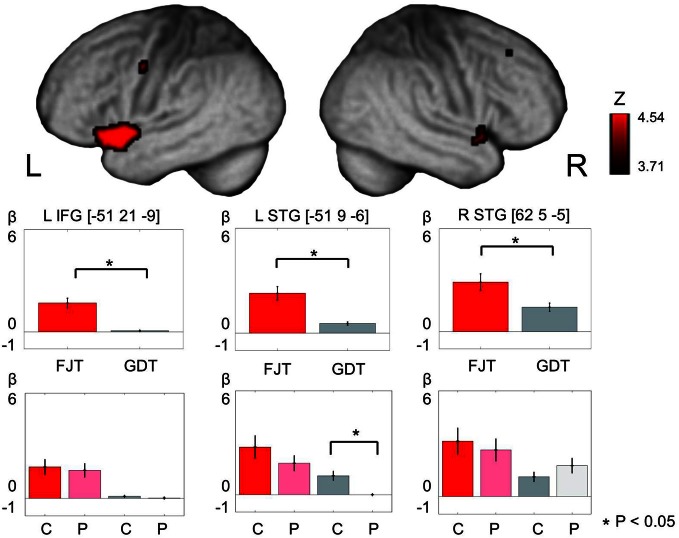
**The figure represents cerebral activation on main effect of task [(FJT/GDT): *p* < 0.05, voxel level, FDR-corrected].** Color bars show the distribution of beta values (mean ± *SD*) under FJT (red) and GDT (gray). ^*^Indicates *p* < 0.05—L, left; R, right; dark color, controls; light color, patients.

Main effect of Task (FJT/GDT) was significantly observed in the left precentral gyrus (PrCG), left MFG, left IFG, right insula, bilateral STG, left claustrum, and left cerebellum (*p* < 0.05, FDR-corrected, Figure 5 and Table 3). Cerebral activation in the left IFG and bilateral STG was significantly greater in FJT than in GDT [Figure 5; L IFG: *t*_(70)_ = 3.92, *p* < 0.05/6; L STG: *t*_(70)_ = 4.64, *p* < 0.05/6; R STG: *t*_(70)_ = 2.92, *p* = 0.005 < 0.05/6]. Interaction effect between Group and Task was not significantly observed at a threshold of *p* < 0.05, FDR-corrected.

**Table 3:** **Peak coordinates (*x*, *y*, *z*) and their *z*-values of cerebral activation by full factorial design analysis with task effect (controls and patients)**.

**Brain regions**	***BA***	**Coordinate**			***F*_(1, 136)_**	***z*-value**	***P* (FDR-corrected)**
		***x***	***y***	***z***			
**MAIN EFFECT OF TASKS (FJT/GDT)**							
L PrCG	6	−50	−4	42	18.50	4.00	<0.05
R MFG	8	27	27	43	16.00	3.70	<0.05
L IFG	47	−51	21	−9	23.90	4.54	<0.05
R insula	13	38	5	−3	19.70	4.13	<0.05
L STG	22	−59	9	−2	30.80	5.13	<0.05
R STG	22	62	5	−5	19.00	4.05	<0.05
L Claustrum		−36	−10	−2	22.20	4.39	<0.05
L cerebellum		−12	−43	−21	20.20	4.13	<0.05

*L, left hemisphere; R, right hemisphere; p < 0.05, voxel level, FDR-corrected*.

### Correlation between psychiatric symptom and cerebral activation

**Figure 6:**
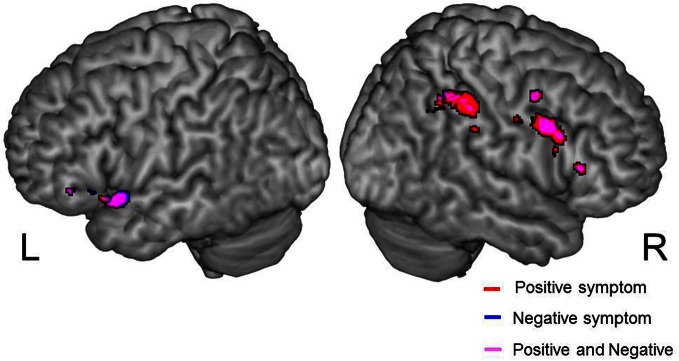
**Positive correlation between psychiatric symptom and cerebral activation under FJT > GDT contrast (univariate analysis).** Positive symptom is red color, Negative symptom is blue color. Purple color indicates both symptoms. Statistical threshold: *p* < 0.25, FDR-corrected; L, left; R, right side.

**Figure 7:**
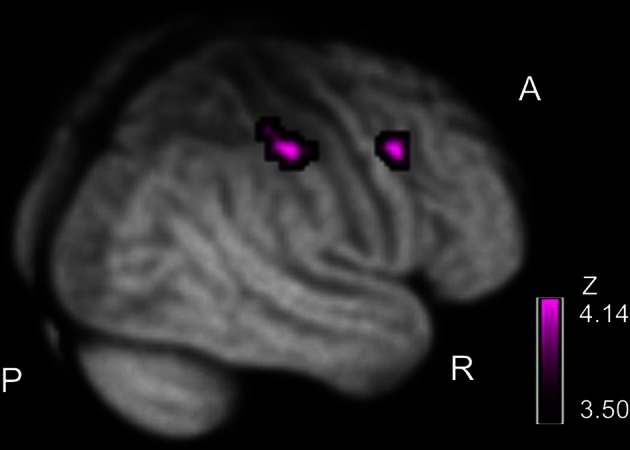
**Positive correlation between severity of hallucinatory behavior and cerebral activation under FJT > GDT contrast.** Purple color bar shows *z*-value. Statistical threshold: *p* < 0.25, FDR-corrected; R, right; A, anterior; P, posterior.

We examined correlations between PANSS and cerebral activation under FJT minus GDT contrast. Significant positive correlations were observed in the right superior frontal gyrus (SFG), right MFG, left IFG, left STG, and right IPL in schizophrenia (*p* < 0.25, FDR-corrected, Figure 6 and Table 4). Figure 7 demonstrated correlations between the severity of auditory hallucinations and cerebral activation under FJT minus GDT contrast. Significant positive correlations were observed in the right post central gyrus (PsCG), right PrCG, right MFG and right IPL in schizophrenia (*p* < 0.25, FDR-corrected, Figure 7 and Table 5).

**Table 4:** **Positive correlation between PANSS and cerebral activation under FJT minus GDT, *p* < 0.0001 uncorrected (*p* < 0.25, FDR-corrected), R, right hemisphere**.

**Brain regions**	***BA***	**Coordinate**			***z*-value**	***P* (uncorrected)**	***P* (FDR-corrected)**
		***x***	***y***	***z***			
L SFG	6	−6	9	55	3.73	<0.0001	<0.25
R SFG	6	9	8	60	4.04	<0.0001	<0.25
R MFG	9	51	6	36	3.77	<0.0001	<0.25
L STG	38	−50	12	−8	3.72	<0.0001	<0.25
R IPL	40	40	−43	49	4.16	<0.0001	<0.25

**Table 5:** **Positive correlation between the severity of hallucinatory behavior and cerebral activation under FJT minus GDT, *p* < 0.0001 uncorrected (*p* < 0.25, FDR-corrected), R, right hemisphere**.

**Brain regions**	***BA***	**Coordinate**			***z*-value**	***P* (uncorrected)**	***P* (FDR-corrected)**
		***x***	***y***	***z***			
**CORRELATION OF HALLUCINATORY BEHAVIOR**							
R PsCG	2	48	−19	30	4.55	<0.0001	<0.25
R PrCG	6	46	−16	30	4.10	<0.0001	<0.25
R MFG	9	48	8	42	3.72	<0.0001	<0.25
R IPL	40	45	−33	46	4.14	<0.0001	<0.25

### Correlation between handedness and cerebral activation

**Figure 8:**
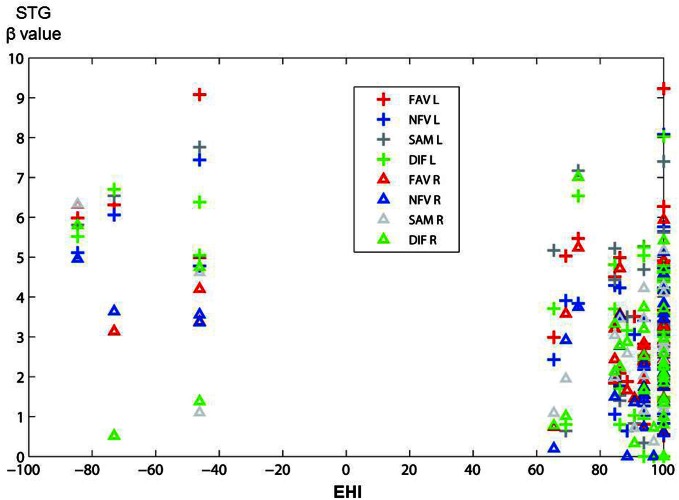
**The figure shows the distribution of correlations between activation in STG (in main effect of Group) and handedness score (EHI) in all participants.** The vertical axis shows beta value in STG, and the horizontal axis shows EHI score (−100 represents extremely left-handed; 100 represents extremely right-handed). Plus marks, left hemisphere; triangles, right hemisphere; FAV (red), NFV (blue), SAM (gray), DIF (green).

We examined the correlation between handedness and cerebral activation. The beta value of ROI analysis in main effect of Group was used in this analysis. Cerebral activations in most ROIs were not correlated with the handedness score, but activation in STG was significantly negatively correlated with the handedness score (Figure 8). In the ROI of STG, differences in LI between 18 controls and 14 patients were analysed after removing 4 left-handed patients. However, significant difference in the LI was not observed.

### Correlation between accuracy of task and cerebral activation

In the ROIs of main effect of Group, correlation was analysed between the beta value of FJT at left STG and accuracy. A significantly positive correlation was observed (*r* = 0.346, *p* < 0.05, Figure 10). The other areas were not significantly correlated with accuracy. These findings suggest that the less the accuracy is, the less the beta value of FJT at left STG is.

## Discussion

**Figure 9:**
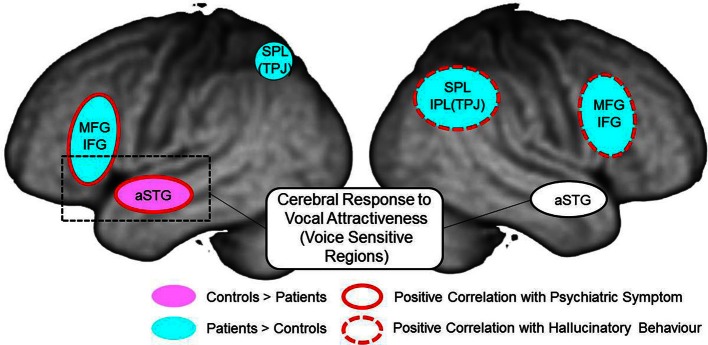
**Summary of cerebral response to auditory attractiveness and psychiatric symptom in schizophrenia: red circle shows the area of positive correlation between cerebral activation and total PANSS score; red dashed line shows the area of positive correlation between cerebral activation and hallucinatory behavior; light blue area represents greater activation in patients than in controls; pink area represents less activation in patients than in controls.** MFG, middle frontal gyrus; IFG, inferior frontal gyrus; aSTG, anterior superior temporal gyrus; SPL, superior parietal lobe; TPJ, temporo-parietal junction; IPL, inferior parietal lobe.

To clarify cerebral function underlying the perception of voice attractiveness including greeting conversations in patients with schizophrenia, we investigated the difference of cerebral activation between control subjects and schizophrenia patients while they were judging favorability or gender of vocalizations. In our present experiment, the left IFG-STG was activated in the processing of favorability judgment in both controls and schizophrenia patients. Although cerebral activation in the left STG was reduced in schizophrenia, cerebral activation in the right MFG, right IPL, and right amygdala was increased. Further, by correlation analysis between psychiatric symptom and cerebral activation of favorability, we confirmed that positive and negative symptoms in schizophrenia are closely related to cerebral dysfunction in the left STG and right MFG-IPL (Figure 9).

**Figure 10:**
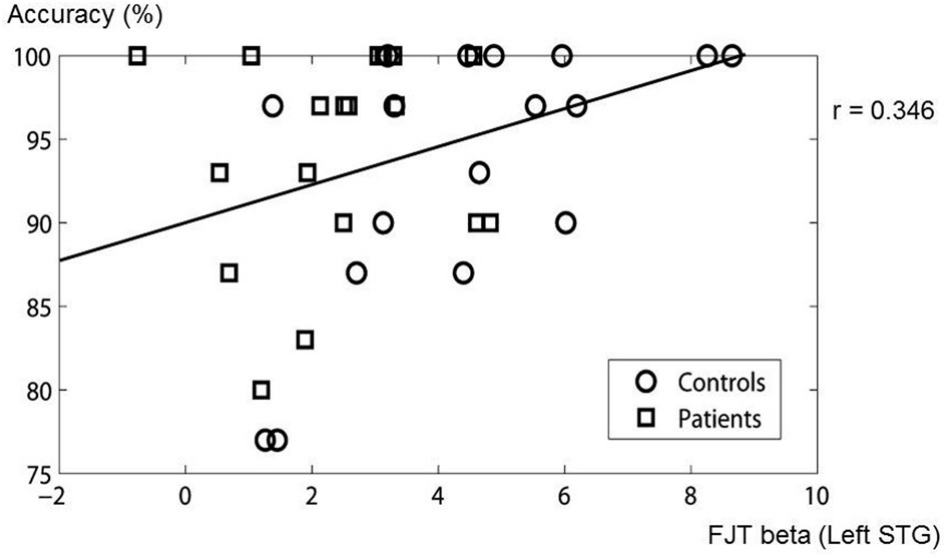
**Correlation between beta value of FJT at left STG and accuracy.** The circles show control subjects. The rectangles show schizophrenia patients.

### Frontotemporal function to auditory attractiveness and its dysfunction in schizophrenia

Our results by full factorial design also exhibited main effect of experimental Task (FJT/GDT) in the left STG and left IFG (Figure 5, Table 3). Recent auditory fMRI studies demonstrated that the cerebral function of STS is important to grasp auditory social cues (Saarela and Hari, 2008; Scharpf et al., 2010). Further, a recent fMRI study concerning auditory attractiveness demonstrated the importance of the functional connection between STG and IFG (Bestelmeyer et al., 2012). In accord with these findings, our results showed left STG-IFG activation in the recognition of auditory attractiveness including social communications.

Before the experiment, we hypothesized that left STG-IFG activation by auditory attractiveness could be impaired in schizophrenia. Predictably, cerebral activation in schizophrenia patients was greater in the bilateral prefrontal regions in comparison with control subjects (Figure 5). A recent study indicated that brain activity in the left prefrontal regions reflected the overall perceived attractiveness of the voices (Bestelmeyer et al., 2012). Further, another fMRI study indicated that the left ventrolateral prefrontal cortex, bilateral dorsal IFG, and medial frontal cortex are activated when the subjects judged whether pairs of human individuals were friends or enemies (Farrow et al., 2011). These reports indicate that prefrontal regions are associated with the judgment of favorability and friendliness. In our present study, hyper-frontality and hypo-temporality in schizophrenia patients could designate the dysfunction of left STG-IFG when they judged favorability.

In our study, cerebral activation to favorability judgment was reduced in the left STG, while it was increased in the MFG, amygdala, TPJ, and occipital lobe in the right hemisphere. A recent fMRI study suggested that paradoxical brain activation in schizophrenia patients with auditory hallucination may be caused by both reduced activation due to impaired brain function in auditory processing and increased activation due to disturbance of attention bias toward internally generated information (Jardri et al., 2011; Kompus et al., 2011). In accordance with this recent study, less activation in schizophrenia could represent impairment of favorability judgment in auditory processing, whereas greater activation in schizophrenia may reflect disturbance of attention bias toward internally generated information by the appearance of auditory hallucination.

### Cerebral laterality to auditory attractiveness in schizophrenia

Interestingly, our results in schizophrenia patients exhibited enhanced right-lateralization to auditory attractiveness mainly in MFG and IPL (Figure 4). Previous fMRI studies concerning language processing have demonstrated that schizophrenia patients show either reduced left hemispheric activation (Kiehl and Liddle, 2001; Kircher et al., 2001; Koeda et al., 2006) or reversed language dominance (Woodruff et al., 1997; Menon et al., 2001; Ngan et al., 2003; Bleich-Cohen et al., 2009). Conversely, previous fMRI studies concerning non-linguistic processing in schizophrenia indicated reduced right hemispheric activation (Koeda et al., 2006), reversed right-lateralized activation (Mitchell et al., 2004), or enhanced right-lateralized activation (Bach et al., 2009). In accordance with the latter report, our results showed greater right prefrontal and inferior parietal activation during favorability judgment in schizophrenia (Figure 4). In the analysis by full factorial design, main effect of Group (controls/patients) revealed greater activation of schizophrenia in the right hemisphere compared with controls (Figure 4). This result also indicates enhanced right hemispheric activation by auditory attractiveness in schizophrenia. It could be speculated that these strong right hemispheric activations compensate the dysfunction of left STG-IFG related to auditory attractiveness (Figure 4).

### Psychiatric symptoms and auditory attractiveness in schizophrenia

Our results revealed a positive correlation between psychiatric symptom (total PANSS score, positive and negative symptom) and cerebral activation under FJT vs. GDT contrast at left STG-IFG and right prefrontal and superior/inferior parietal cortex (Figure 6 and Table 4). In both positive and negative symptoms, almost the same regions were correlated with cerebral activation for auditory attractiveness. Left STG-IFG activation was observed in the favorability judgment (Figure 5). These findings could be considered to reflect the dysfunction of the left STG-IFG region in the recognition of auditory attractiveness. Crucially, cerebral activation in the right prefrontal and superior/inferior parietal region was positively correlated with the severity of auditory hallucination (Figure 7, Table 5). These areas also demonstrated greater activation under FJT vs. GDT contrast in schizophrenia (Figure 5, Table 3). These findings indicate that greater activation to the favorability judgment in schizophrenia is related to severity of auditory hallucinations. Previous studies indicate that the right MFG/IFG-IPL region is closely related to self-referential processing (Fossati et al., 2003; Canessa et al., 2005; Uddin et al., 2005). Especially, one study demonstrated that right fronto-parietal regions as well as left prefrontal and parietal regions were activated when subjects understood the context related to social communications when two persons exchange goods, i.e., if you give me one, I will give you the other (Canessa et al., 2005). Further, another study exhibited that right dorsal IFG was activated in the processing of social alliance (friendliness) (Farrow et al., 2011). These previous findings support that the right MFG/IFG-IPL region associates with the recognition of social communications such as judgment of favorability. These activations could be attributed to representing the dysfunction of the fronto-parietal region in the processing of social communications by auditory hallucinations.

Recent fMRI studies investigated cerebral function when the subjects mentalize the other person's thoughts and behavior. These reports indicate that the role of the temporal-parietal junction is closely associated with comprehending the mental states of others (Siegal and Varley, 2002; Finger et al., 2006; Shamay-Tsoory et al., 2006; David et al., 2008). A recent study investigated cerebral activation in the processing of self-other distinction. This study demonstrated that the increase in cerebral activation in the right IPL correlated positively with the strength of psychiatric symptoms in schizophrenia (Jardri et al., 2011). Further, recent studies of schizophrenia reported that functional connectivity in the fronto-temporal network was decreased when the subjects comprehended the behavior of the other person (Das et al., 2012), or when the subjects listening to the other person's speech compared it with self-generated speech (Mechelli et al., 2007). Findings of greater right prefrontal-parietal activation (Figure 4) in schizophrenia may reflect brain activation due to comprehending other person's mental states through auditory hallucination as well as dysfunction of the fronto-temporal region in perception of vocal attractiveness.

In summary, when cerebral function in auditory attractiveness including social conversations was investigated, cerebral activation was revealed in the left STG and left IFG. Particularly, in schizophrenia, less activation was observed at the left STG compared with control subjects. In addition, greater activation in schizophrenia was confirmed in the right fronto-parietal region. Further, cerebral response in this region was correlated with the severity of auditory hallucinations. These findings suggest that dysfunction in the left fronto-temporal regions is related to the ability to appropriately assess the attractiveness of vocal communications in schizophrenia. The right fronto-parietal region could offset cerebral dysfunction to auditory attractiveness including social communications.

### Conflict of interest statement

The authors declare that the research was conducted in the absence of any commercial or financial relationships that could be construed as a potential conflict of interest.
